# Prevention of Disuse Muscle Atrophy by Dietary Ingestion of 8-Prenylnaringenin in Denervated Mice

**DOI:** 10.1371/journal.pone.0045048

**Published:** 2012-09-19

**Authors:** Rie Mukai, Hitomi Horikawa, Yutaka Fujikura, Tomoyuki Kawamura, Hisao Nemoto, Takeshi Nikawa, Junji Terao

**Affiliations:** 1 Department of Food Science, Institute of Health Biosciences, University of Tokushima Graduate School, Tokushima, Japan; 2 Department of Pharmaceutical Chemistry, Institute of Health Biosciences, University of Tokushima Graduate School, Tokushima, Japan; 3 Department of Nutritional Physiology, Institute of Health Biosciences, University of Tokushima Graduate School, Tokushima, Japan; Istituto di Ricerche Farmacologiche Mario Negri, Italy

## Abstract

Flavonoids have attracted considerable attention in relation to their effects upon health. 8-Prenylnaringenin (8-PN) is found in the common hop (*Humulus lupulus*) and assumed to be responsible for the health impact of beer consumption. We wanted to clarify the effects of prenylation on the physiological functions of dietary flavonoids by comparing the effects of 8-PN with that of intact naringenin in the prevention of disuse muscle atrophy using a model of denervation in mice. Consumption of 8-PN (but not naringenin) prevented loss of weight in the gastrocnemius muscle further supported by the lack of induction of the protein content of a key ubiquitin ligase involved in muscle atrophy, atrogin-1, and by the activation of Akt phosphorylation. 8-PN content in the gastrocnemius muscle was tenfold higher than that of naringenin. These results suggested that, compared with naringenin, 8-PN was effectively concentrated into skeletal muscle to exert its preventive effects upon disuse muscle atrophy. It is likely that prenylation generates novel functions for 8-PN by enhancing its accumulation into muscle tissue through dietary intake.

## Introduction

Flavonoids are plant secondary metabolites derived from malonyl-CoA and *p*-coumaroyl-CoA. Flavonoids have attracted considerable attention in relation to their effects upon human health [1], [2]. In prenylflavonoids, the C_5_ isoprenoid group is substituted in the diphenylpropane structure. Prenylflavonoids such as prenylchalcones, prenylflavones, prenylflavonols and prenylflavanones are distributed mainly in the roots, leaves, and seeds [3], [4]. In particular, prenylflavanones occur mostly in Legmuminosae, Moraceae and Asteraceae [3]. Recent studies have suggested that prenylflavonoids exert powerful biological functions. For example, the prenylflavonol icaritin was shown to prevent cell growth by inducing cell-cycle arrest in carcinoma cells [5]. Prenylflavanones extracted from the roots of *Sophora flavescens* were found to possess antibacterial and anti-androgen activities [6]. Prenylflavones from *Psoralea corylifolia* and *Mori Cortex Radics* suppressed the production of nitric oxide in nerve cells [7], [8]. Prenylation has been demonstrated to enhance the estrogenic activity of (1) naringenin [9] and the tyrosinase activity of luteolin [10]. These observations suggest the superiority of prenylflavonoids to non-prenylflavonoids within *in vitro* cell culture systems. However, little is known about the effect of prenylation on the physiological functions and bioavailability of dietary flavonoids *in vivo*. Recently, Sasaki et al. [11] discovered flavonoid prenyltransferase from the legume *Sophora flavescens*, and successfully undertook molecular cloning of this enzyme. That report urged us to explore a novel physiological function of prenylflavonoids because various prenylflavonoids seem to be readily prepared using this enzyme.

**Figure 1:**
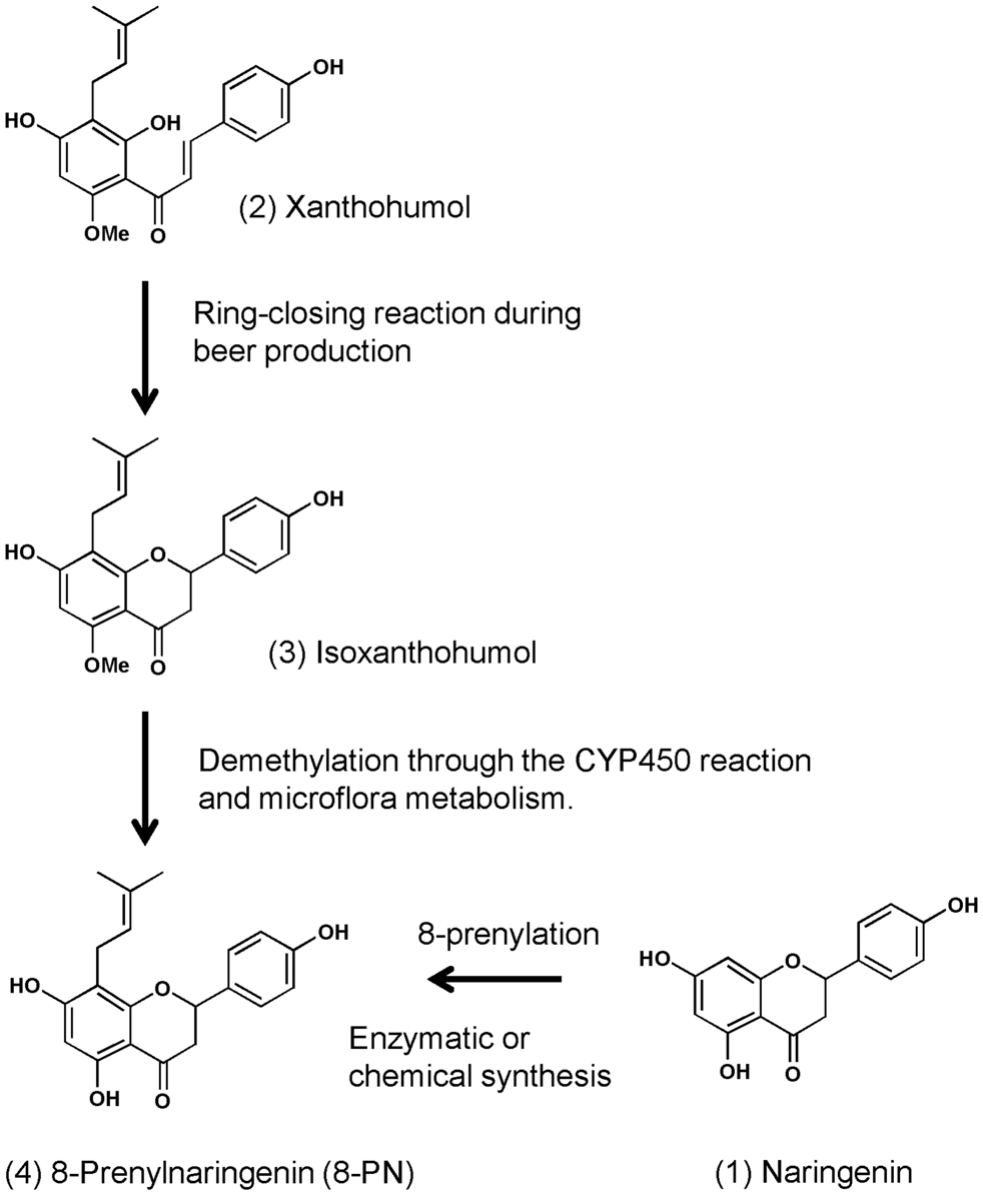
Conversion of xanthohumol to 8-prenylnaringenin (8-PN).

A prenylchalcone, (2) xanthohumol, is the principal flavonoid in the common hop (*Humulus lupulus*) [12]. In beer production, xanthohumol undergoes a ring-closing reaction, resulting in the formation of (3) isoxanthohumol [13]. This prenylflavonoid is further converted to (4) 8-prenylnaringenin (8-PN) by demethylation of the methoxy group attached to the diphenylpropane structure through the hepatic CYP450 reaction [14] and microflora metabolism in humans [15], [16] (Figure 1). Therefore, humans may ingest 8-PN as a metabolite of isoxanthohumol as well as a hop ingredient in beer. Animal studies have revealed that a prenylflavanone, 8-PN, exerts beneficial effects against hot flushes [17] and bone loss [18]. Prenylation at the 8-position of naringenin was found to be responsible for the enhanced estrogenic activity of 8-PN in a rat serotonergic cell line [19]. These findings suggested that prenylation plays an important part in the physiological function of dietary flavonoids. Nevertheless, how prenylation enhances the physiological function of dietary flavonoids in the body is not known.

The aim of the present study was to compare the effect of 8-PN (prepared by chemical synthesis [20]) with that of non-prenylated naringenin (which is commonly found in grapefruit and sour oranges [21]) on disuse muscle atrophy using a denervation model in mice. This was done because estrogenic activity has been reported to be associated with muscle maintenance by activating Akt phosphorylation [22], [23]. Denervation induces expression of atrogin-1 (a skeletal muscle-specific ubiquitin ligase) and mice deficient in atrogin-1 are resistant to muscle atrophy caused by denervation [24], [25]. Phosphorylation of Akt has been suggested to suppress the expression of atrogin-1 by preventing activation of the transcription factor for atrogin-1: the Forkhead box-containing, O subfamily (FoxO) family of transcription factors [26], [27], [28].

We also examined the concentrations of 8-PN and naringenin in blood plasma and target muscle tissues to evaluate the effect of prenylation on the bioavailability and liability for transport to the target site in the prevention of muscle atrophy by dietary flavonoids. The results of the present study were expected to be helpful in estimation of the effects of brewery products on human health.

## Materials and Methods

### Materials

Naringenin was obtained from Tokyo Chemical Industry, Tokyo, Japan). 8-PN was synthesized by our research team [20]. Dried *H. lupulus* powder was obtained from Nippon Funmatsu Yakuhin (Osaka, Japan).

### Animal Experiments

All experimental protocols were in accordance with the guidelines for the care and use of laboratory animals set by the Graduate School of the Institute of Health Biosciences, the University of Tokushima (Tokushima, Japan). The protocol was approved by the Committee on Animal Experiments of the University of Tokushima (permit number: 11013). All surgery was undertaken under anesthesia using sodium pentobarbital or ether. All efforts were made to minimize the suffering of the animals.

**Table 1:** Protein and water content in the GM.

Diet	Condition	Protein content(mg/g GM)	Water content(%, w/w)
Control	Sham	61.7±2.4	76.2±0.29
	Denervation	65.4±0.9	75.8±0.52
PN	Sham	63.1±1.6	76.3±0.52
	Denervation	66.9±2.6	77.3±0.37
N	Sham	59.1±2.1	76.9±0.71
	Denervation	62.9±1.6	77.9±0.39

Data are the mean ± S.E. There was no significant difference upon analyses by the Tukey multiple comparison test with one-way ANOVA (*p*<0.05).

### Experiment I: Estimation of Loss in Muscle Weight

Seven-week-old male C57/BL6 mice (Japan SLC, Shizuoka, Japan) were housed in a room maintained at 23±1°C on a 12-h light–dark cycle. They were allowed free access to a commercial diet (AIN-93M; Oriental Yeast Company, Tokyo, Japan) and water. The sciatic nerve in the right leg of each mouse was cut to induce immobilization, and then disuse muscle atrophy provoked in the gastrocnemius muscle (GM) (i.e., denervation). Sham surgery was undertaken in the left leg of each mouse to obtain control muscle. The GM was collected and its weight measured 2, 4, and 6 days later.

**Figure 2:**
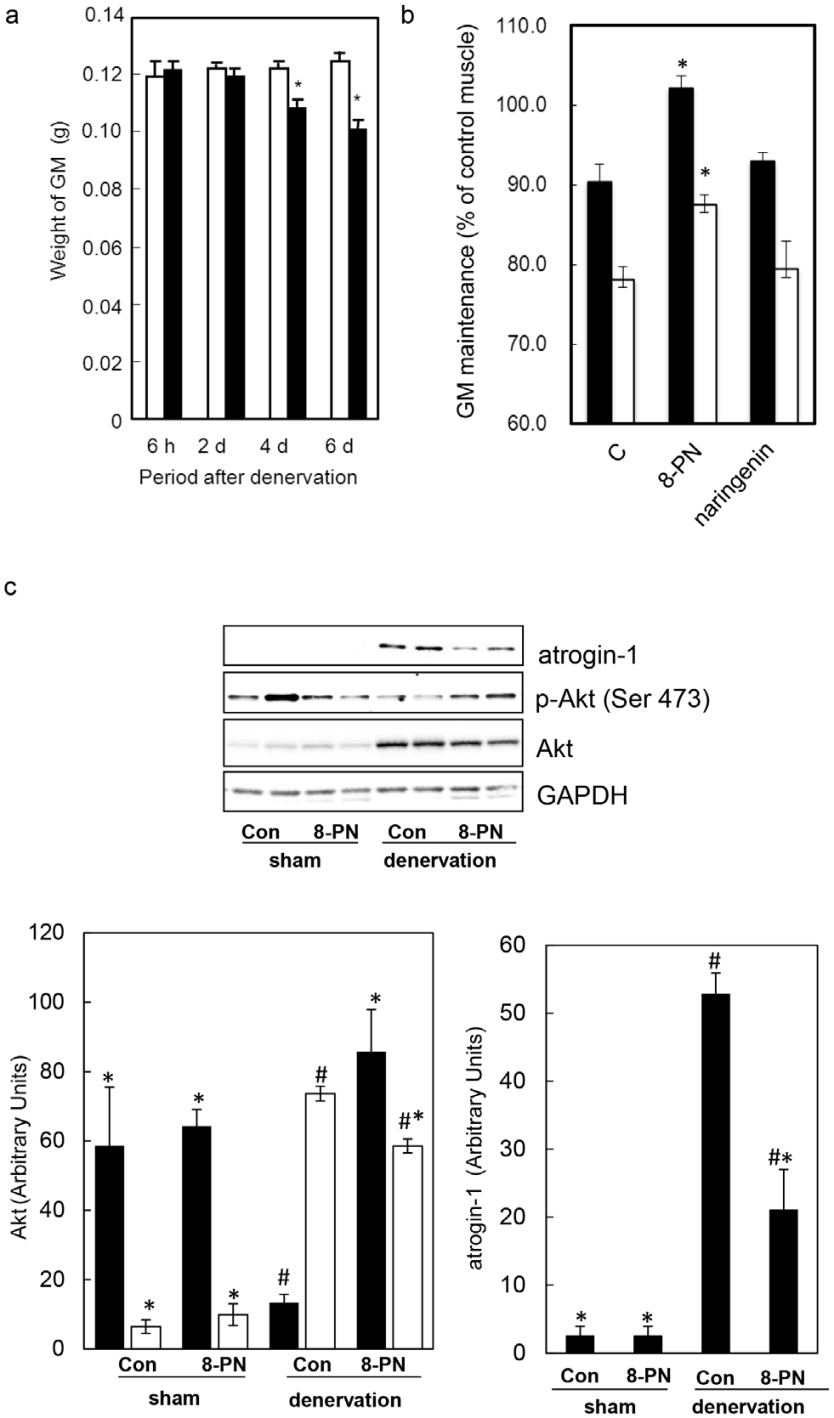
8-PN can prevent disuse muscle atrophy by enhancing Akt phosphorylation. (a) Muscle atrophy induced by denervation. The weight of the GM was measured after denervation for the indicated period. Open bar: sham leg (left); closed bar: denervated leg (right). Data are the mean ± S.E (n = 4). Asterisks indicate significant differences between sham and the denervated leg (Student’s *t*-test, *p*<0.007). (b) Effect of dietary intake of 8-PN or naringenin on muscle atrophy. Mice consumed each flavonoid-mixed diet for 18 days, and denervation was then carried out. After 4 (black bar) or 6 (white bar) days, the level of atrophy in the GM was calculated as the ratio of the weight of denervated muscle to the weight of sham muscle in each mouse. Data are the mean ± S.E (n = 4). C: control-diet group, 8-PN: 8-PN-containing diet group. Asterisks indicate significant differences to the control diet, which was analyzed by the Tukey multiple comparison test with one-way ANOVA (day 4: *p* = 0.0034; day 6: *p* = 0.041). (c) Phosphorylation of Akt and atrogin-1 in the GM (which was collected on the 6th day after denervation) was detected by western blotting (upper) and the density of each image analyzed (bottom). The black bar and white bar in left graph denote phosphorylated Akt and total Akt, respectively. Data are the mean ± S.E (n = 4). C: control-diet group, 8-PN: 8-PN-containing diet group. *Significant differences to the control diet-denervation group (*p*0.05). #Significant differences to the control diet-sham group.

### Experiment II: Preventive Effects of Continuous Intake of 8-PN and Naringenin on Disuse Muscle Atrophy

8-PN or naringenin (5.6 mmol/kg diet) was mixed with AIN-93M and given to mice. Cellulose content was reduced to adjust for the composition of other nutrients. Denervation was carried out on day 18. The GM was collected 4 or 6 days later. The level of atrophy in the GM was calculated to be the ratio of the weight of denervated muscle to the weight of control muscle in each mouse. Samples of the GM were stored at –80°C under N_2_ gas until high-performance liquid chromatography (HPLC) analyses. The GM was immediately used for the measurement of protein and water contents.

**Figure 3:**
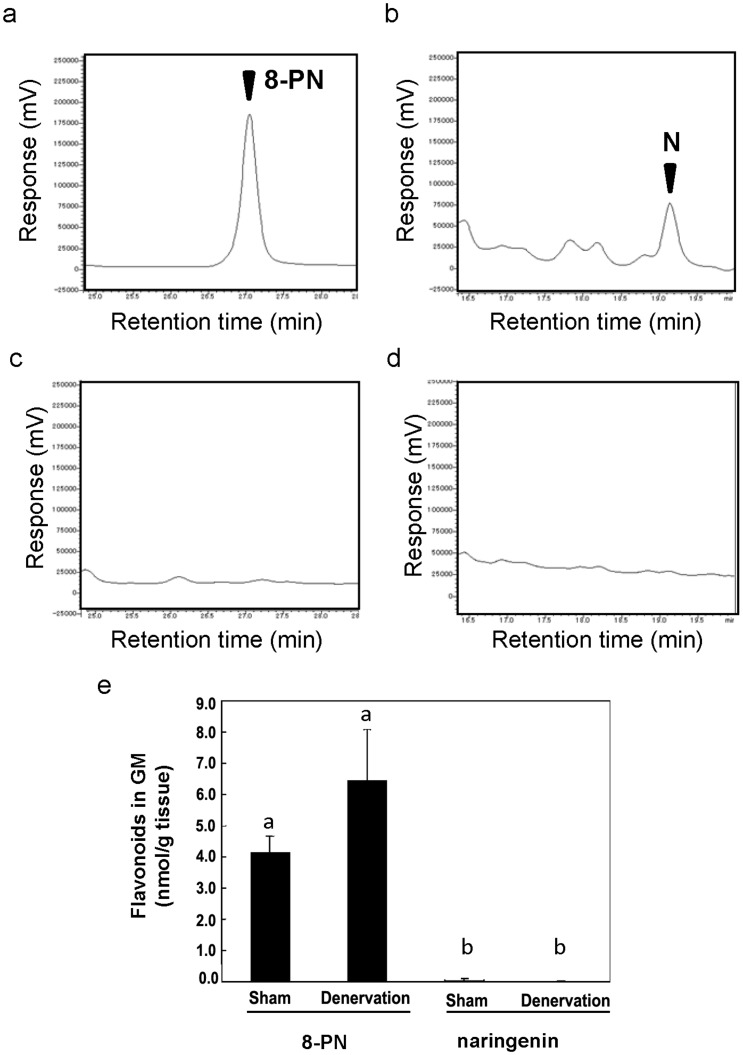
Accumulation of 8-PN in the GM. (a)–(e): HPLC chromatograms for quantitative analyses of 8-PN or naringenin in the GM. Chromatograms from mice fed an 8-PN-containing diet (a) and control diet (b). Chromatograms from mice fed a naringenin-containing diet (c) and control diet (d). (a) and (b) were obtained by the analytical condition for 8-PN. (c) and (d) were obtained by the analytical condition for naringenin. These analyses were undertaken by HPLC with electrochemical detection. (e) Contents of these flavonoids in the GM as determined by HPLC analysis. Data are the mean ± S.E (n = 4). Different letters indicate significant differences analyzed by the Tukey multiple comparison test with two-way ANOVA (*p* = 0.00043).

### Experiment III: Accumulation of 8-PN in the GM and Plasma after Dietary Consumption

Experimental conditions (mice, feeding, denervation) until sample collection were identical to those described in experiment II. Blood collection and body reflux was performed before collection of the GM. Plasma was isolated from blood by centrifugation at 9,000×*g* for 10 min at 4°C. Samples of the GM and plasma were stored at –80°C under N_2_ gas until HPLC analyses.

**Table 2:** Accumulation of 8-PN and naringenin in the GM and plasma after their dietary intake for 22 days.

	8-PN		Naringenin	
*GM*	Sham	Denervation	Sham	Denervation
Total (nmol/g tissue, n = 7)	2.26±0.33^ a^	3.00±0.30^a^	0.26±0.04^b^	0.23±0.02^b^
Aglycone (nmol/g tissue, n = 6)	0.35±0.09	0.50±0.06	Not determined	Not determined
*Plasma*				
Total (µM, n = 7)	38.5±2.72		Not detected ≤0.025	
Aglycone (µM, n = 7)	0.06±0.01		Not detected ≤0.025	

Blood was collected from mice fed flavonoid (5.6 mmol flavonoid/kg diet) for 22 days, and then total body reflux was carried out. The GM was collected after the reflux. Plasma was prepared from the blood as described in the Materials and Methods section. Values of the total 8-PN and total naringenin indicate the sum of aglycone and conjugated metabolites obtained by the HPLC analysis with deconjugation treatment. Amount of flavonoids was determined by HPLC analyses as described in the Materials and Methods section. Data are the mean ± S.E. Different letters indicate significant differences upon analyses by the Tukey multiple comparison test with two-way ANOVA (*p* = 1.79×10^–9^).

### Experiment IV: Pharmacokinetics of 8-PN and Naringenin

Seven-week-old male C57/BL6 mice (Japan SLC) were housed in a room maintained at 23±1°C on a 12-h light–dark cycle. They were allowed free access to a commercial diet (AIN-93M) and water for 1 week. Before administration, they were deprived of food for 18 h, but had free access to water. 8-PN or naringenin dissolved in propylene glycol was administered (50 mg/kg body weight (bw)) to mice by a gastric feeding tube. Blood was collected 0.25, 0.5, 1, 2, 4, 8, and 24 h after administration. Plasma was isolated by centrifugation at 9,000×*g* for 10 min at 4°C and stored at –80°C under N_2_ gas until use.

**Figure 4:**
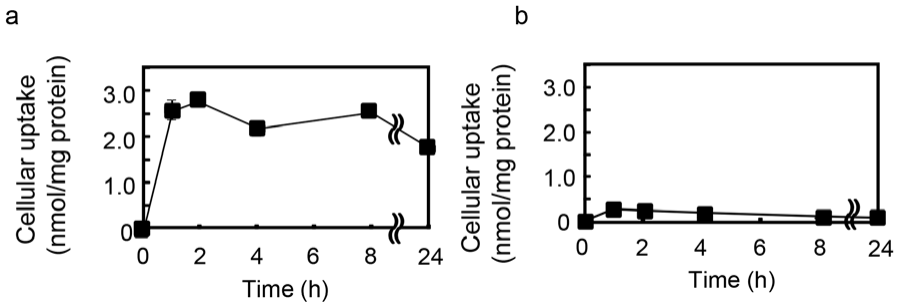
Cellular uptake of 8-PN in mouse C2C12 myotubes. Differentiated C2C12 cells seeded on 60-mm dishes were used. (a) 8-PN and (b) naringenin (10 µM) were administered to the cells for the indicated time. Cell homogenates were prepared and then each flavonoid was extracted. Amounts of flavonoids were determined by HPLC with UV detection. Data are the mean ± S.E (n = 3).

### Experiment V: Preventive Effect of *H. lupulus* on Disuse Muscle Atrophy

Dried *H. lupulus* powder was mixed (5% *w/w*) with AIN-93M and given to mice. Cellulose content was reduced to adjust for the composition of other nutrients. Denervation was carried out on day 14. The GM was collected 4 days later. The level of atrophy of the GM was calculated to be the ratio of the weight of denervated muscle to the weight of control muscle in each mouse.

**Table 3:** Pharmacokinetic parameters of flavonoids after oral administration of 8-PN and naringenin (50 mg/kg body weight) in a single dose in mice.

Parameter	8-PN	Naringenin
C_max_ (µM)	28.7±3.05	129±34.0*
AUC (µmol⋅h/L)	101±11.4	199±18.2*
T_max_ (h)	0.25±0	0.31±0.063
T_1/2_ (h)	0.86±0.26	0.55±0.26
Concentration at 24 h (µM)	2.6±1.5	0.46±0.28

C_max_: maximum concentration in plasma; AUC: area under the plasma concentration–time curve; Tmax: time to maximum plasma concentration; T_1/2:_ half-life of flavonoid in the elimination phase.

Each flavonoid (50 mg/kg bw) was administered to mice once by stomach intubation. Plasma concentration was analyzed by HPLC–UV. Data are the mean ± S.E (n = 4). Asterisks indicate significant differences between two groups (C_max_: *p* = 0.026; AUC: *p* = 0.0039, Student’s *t-*test).

**Figure 5:**
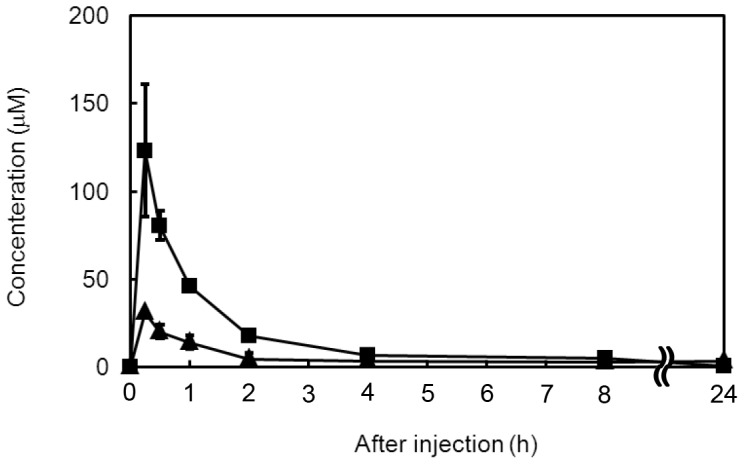
Plasma concentration of 8-PN or naringenin after their oral administration in mice. Each flavonoid was orally administered at 50 mg/kg bw in a single dose by stomach intubation. The plasma concentration of each flavonoid was determined by HPLC–UV after deconjugation treatment. Closed triangle: 8-PN, closed square: naringenin. Data are the mean ± S.E (n = 4).

### Measurement of Water Content and Protein Concentration in the GM

Water content in muscle was determined by measuring dry muscle weight [30]. About 50 mg of the wet mass of the GM was weighed, subjected to a drying heater at 70°C, and weighed every 60 min until a constant dry weight was obtained.

**Figure 6:**
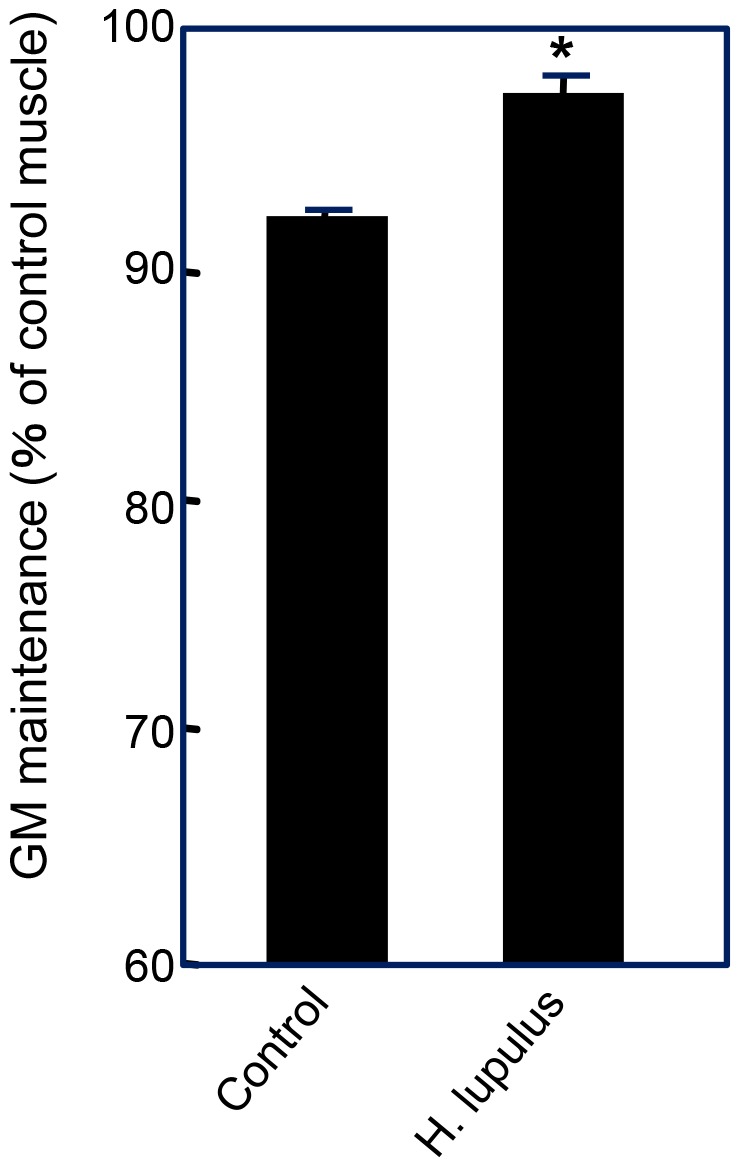
Preventive effect of *Humulus lupulus* on disuse muscle atrophy. Mice consumed a *Humulus lupulus*-mixed diet for 14 days, after which denervation was carried out. After 4 days, the weight of the GM was measured. The level of atrophy was calculated to be the ratio of the weight of denervated muscle to the weight of sham muscle in each mouse. Data are the mean ± S.E (n = 3). Asterisks indicate significant differences analyzed by the Student’s *t*-test (P = 0.0046).

For the measurement of protein concentration, the GM was homogenized on ice in buffer containing 50 mM HEPES (pH 7.4), 4 mM ethylene glycol tetra-acetic acid (EGTA), 20 mM ethylene diamine tetra-acetic acid (EDTA), 0.1% Triton X-100, a protease inhibitor tablet (Complete; Roche Applied Science, Indianapolis, IN, USA) and a phosphatase inhibitor tablet (PhosSTOP; Roche Applied Science). Samples were incubated on ice for 1 h with shaking every 10 min. Samples were centrifuged at 20,000×*g* for 20 min at 4°C. The supernatant was collected and protein concentration determined in triplicate using the Bradford method [31].

### Western Blotting

The GM was homogenized on ice in buffer containing 50 mM Tris-HCl (pH 7.5), 150 mM NaCl, 5 mM EDTA, 1% Triton X-100, a protease inhibitor tablet (Complete) and a phosphatase inhibitor tablet (PhosSTOP). Samples were incubated on ice for 1 h with shaking every 10 min. Samples were centrifuged at 20,000×*g* for 20 min at 4°C. Supernatants were collected as whole protein lysates. The protein concentration was determined in triplicate using the Bradford method [31]. Protein lysates were separated by 10% sodium dodecyl sulfate-polyacrylamide gel electrophoresis (SDS-PAGE). Proteins were transferred onto polyvinylidene difluoride membranes (GE Healthcare Piscataway, NJ, USA) followed by blocking of non-specific binding with a commercial blocking buffer (Blocking-One, Nacalai Tesque, Kyoto, Japan) for 1 h. Membranes were incubated with anti-atrogin-1 antibody (1∶1,000 dilution; ECM Biosciences, Versailles, KY, USA) anti-pAKT^ser473^ antibody (1∶500; Cell Signaling Technology, Danvers, MA, USA), anti-pAKT^thr308^ antibody (1∶500; Cell Signaling Technology), anti-glyceraldehyde 3-phosphate dehydrogenase (GAPDH) antibody (1∶5,000; Cell Signaling Technology), and ant-Akt antibody (1∶1,000; Cell Signaling Technology) for 1 h at room temperature. After washing with Tris-buffered saline containing 0.05% Tween 20 (TBST), membranes were incubated with peroxidase-labeled secondary antibody for 1 h. After washing three times with TBST (for pAkt) or phosphate-buffered saline containing 0.05% Tween 20 (PBST) (for other proteins), immunocomplexes were visualized using a Chemiluminescence Detection Kit (ECL Prime, GE Healthcare BioScience) and analyzed by Image J software (National Institutes of Health, Bethesda, MD, USA).

### Analyses of 8-PN and Naringenin in the GM

Each GM was homogenized with phosphate-buffered saline (PBS; 9-times volume of the weight of GM) using a Teflon Homogenizer (As One, Osaka, Japan) on ice. For analyses of total 8-PN or naringenin (conjugated metabolites plus aglycone) in the GM, muscle homogenates were mixed with 50 mM ascorbic acid (0.2-times volume of PBS) and 100 U/0.1 mL β-glucuronidase type H-1 solution (final concentration: 9 units/mg GM; Sigma–Aldrich, St Louis, MO, USA) and incubated for 2 h at 37°C. Hydrolysates were extracted three times with the same volume of ethyl acetate and evaporated using a Centrifugal Evaporator (CVE-100, Tokyo Rikakikai, Tokyo, Japan). Extracts were dissolved in 150 µL (for 8-PN) or 30 µL (for naringenin) of methanol containing 0.5% phosphoric acid. Twenty microliters of the sample were injected into the HPLC electrochemical detection system (700 mV; Coulochem III, ESA, Cambridge, MA, USA) equipped with a Cadenza 3-µm CD-C18 HPLC column (150×4.6 mm; Imtakt, Hampshire, UK). Separation of compounds was carried out by gradient elution. Solvent A was 0.5% phosphoric acid, and solvent B was acetonitrile containing 0.5% phosphoric acid. For 8-PN detection, the gradient program was: 0 min, 30% B; 0–15 min, linear gradient to 40% B; 15–35 min, linear gradient to 60% B; 35–36 min, linear gradient to 100% B; 36–39 min, 100% B; 39–40 min, linear gradient to 30% B; flow rate, 1 mL/min. For naringenin detection, the gradient program was: 0 min, 15% B; 0–10 min, linear gradient to 33% B; 15–25 min, linear gradient to 60% B; 25–26 min, linear gradient to 100% B; 26–30 min, 100% B; 30–31 min, linear gradient to 15% B; 31–35 min, 15% B; flow rate, 1 mL/min.

### Cell Culture and Sample Preparation for Cellular Uptake

The mouse myoblast cell line C2C12 (American Type Culture Collection, Rockville, MD, USA) was maintained in Dulbecco’s modified Eagle’s medium (DMEM) supplemented with 10% fetal bovine serum, 100 U/mL penicillin, 100 µg/mL streptomycin, and 2 mM L-glutamine at 37°C in a humidified atmosphere containing 5% CO_2_. Cells seeded on a 60-mm dish were cultured until confluent. Differentiation was initiated with 2% horse serum containing DMEM for 96 h. Cells were incubated with 10 µM 8-PN or naringenin. After incubation, cells were washed twice with ice-cold PBS and scraped from the dish. The protein concentration of cell suspensions was measured using the Bradford assay [31]. After centrifugation (9,000×*g* for 10 min at 4°C) 8-PN and naringenin were extracted three times in ethyl acetate by sonication for 1 min using an Astrason XL2020 Ultrasonic Processor (Heat Systems-Ultrasonic, Farmingdale, NY, USA) at level 10. Before extraction, 100 pmol of pentamethyl quercetin (for 8-PN) or kaempferol (for naringenin) was added to cell suspensions as the internal standard. After centrifugation (9,000×*g* for 10 min at 4°C), supernatants were collected, evaporated under N_2_ gas, and dissolved in 50 µL of methanol containing 0.5% phosphoric acid. Twenty microliters of the sample were injected into the HPLC–UV system.

### Sample Preparation for the Determination of Plasma Concentration

To analyze the concentration of metabolites and/or aglycone, plasma (10 µL) was incubated with 100 U of β-glucuronidase type H-1 (which possessed β-glucuronidase and sulphatase activity) in 0.1 M sodium acetate buffer, pH 5.0 (90 µL) and 50 mM ascorbic acid (20 µL) for 45 min. To measure the aglycone concentration, plasma without deconjugation treatment was applied to the experiment. Then, 100 pmol of pentamethyl quercetin (for 8-PN) or kaempferol (for naringenin) was added to samples as the internal standard. The liberated aglycone was extracted with ethyl acetate and evaporated using the Centrifugal Evaporator, and dissolved in 75 µL of methanol containing 0.5% phosphoric acid. A total of 25 µL of the sample was injected into the HPLC–UV detection system.

### Calculation of *C*logP Values

ClogP values of 8-PN and naringenin were calculated using ChemBioDraw Ultra ver11.0 (PerkinElmer, Waltham, MA, USA).

### Determination of Cellular Uptake and Plasma Concentration

8-PN and naringenin were analyzed by HPLC–UV detection using a λ_max_ value of 292 nm (SPD-10AV; Shimadzu, Tokyo, Japan) equipped with a TSK-gel ODS-80Ts HPLC column (150×4.6 mm; Tosoh, Tokyo, Japan). In the mobile phase, solvent A was 0.5% phosphoric acid, and solvent B was methanol containing 0.5% phosphoric acid. For 8-PN detection, B was 65%. For naringenin detection, B was 55%. The flow rate was 1.0 mL/min.

### Statistical Analyses

Data are the mean ± SE. Data of protein content and water content in the GM, effect of dietary intake on 8-PN or naringenin on muscle atrophy and phosphorylation of Akt and atrogin-1 were analyzed by one-way ANOVA with the Tukey multiple comparison test (p<0.05). Data of accumulation of 8-PN or naringenin in the GM and plasma were analyzed by two-way ANOVA with the Tukey multiple comparison test (p<0.05). Data of the effect of denervation on muscle atrophy, effect of *H. lupulus* on muscle atrophy as well as pharmacokinetic parameters of 8-PN and naringenin were analyzed by the two-sided Student's t-test (p<0.05).

## Results

### Preventive Effects of 8-PN on Disuse Muscle Atrophy

Denervation was initiated in the right legs of mice to induce loss of muscle weight in this model of disuse muscle atrophy [32]. The body weight of mice did not change ≤6 days after denervation (data not shown). In contrast, the weight of the GM of the denervated leg was significantly decreased 4 days and 6 days after denervation (Figure 2a). Next, mice were fed a diet containing 8-PN or naringenin (5.6 mmol flavonoid/kg diet) for 18 days before denervation. After denervation, these mice were fed the same diet for an additional 4 days or 6 days. Collection of the GM and blood was carried out just after the end of feeding period (22 days or 24 days). No significant difference was observed in food intake among the control-diet group, 8-PN-containing diet group or naringenin-containing diet group during the experiment (daily consumption of 8-PN and naringenin was 1150–1250 nmol flavonoid/kg bw/day). Figure 2b shows that 8-PN intake significantly suppressed the denervation-induced loss in muscle weight, whereas naringenin intake did not elicit such an effect. 8-PN and naringenin did not attenuate the normal muscle mass (as % of body weight: control diet, 0.57±0.01; PN diet, 0.55±0.02; naringenin diet, 0.58±0.01; mean±SE, n = 4). With respect to water and protein contents, there were no differences among all groups (Table 1). This finding suggested that GM content was affected by 8-PN-intake as well as denervation. These findings suggested that the prenyl group was essential for 8-PN to exert its preventive effects on loss in muscle weight.

The phosphorylation of residue ser-473 on Akt was measured to ascertain whether 8-PN affects the balance of synthesis and degradation of proteins in the inhibition of disuse muscle atrophy (Figure 2c). The GM 6 days after denervation was applied for western blotting. Total Akt expression was increased by denervation, and 8-PN intake showed no effect on the expression. Although Akt phosphorylation was lowered by denervation, 8-PN intake retained the phosphorylation status on Akt in denervated muscle similar to that seen with sham muscle. No differences were observed in GAPDH contents among all groups. We also detected atrogin-1 in the GM. Denervation increased the level of atrogin-1. Intake of 8-PN suppressed the atrogin-1 level in the denervated muscle, suggesting that 8-PN intake delayed the development of disuse muscle atrophy due to suppression of protein degradation.

### Accumulation of 8-PN in the GM

The contents of 8-PN and naringenin in the GM were determined by HPLC analyses with electrochemical detection employing deconjugation treatment using β-glucuronidase (which also had sulphatase activity) (Figures 3a–d). Peaks attributable to 8-PN and naringenin appeared in the chromatogram of the extract from the GM of mice fed diets containing 8-PN and naringenin, respectively (Figures 3a and c). The retention times of these peaks coincided with those for the standards of 8-PN and naringenin. No peaks appeared at the corresponding retention times in the chromatogram from mice fed the control diet (Figures 3b and d), indicating that both flavonoids could accumulate in GM tissue by oral intake of 8-PN and naringenin. Total amounts of flavonoids (including both aglycones and their conjugated metabolites) could be obtained by deconjugation of the extracts. The content of total 8-PN was 4.12±0.56 nmol/g tissue (sham: without denervation) and 6.44±1.65 nmol/g tissue (with denervation). The content of total naringenin was approximately tenfold lower than that of total 8-PN: 0.56±0.04 nmol/g tissue (sham: without denervation) and 0.070±0.02 nmol/g tissue (with denervation) (Figure 3e). We further determined the contents of total flavonoids and their aglycones in GM. To avoid blood contamination in the GM, we carried out a body reflex before GM collection (experiment III). The content of total 8-PN was 2.26±0.33 nmol/g tissue (sham: without denervation) and 3.00±0.30 nmol/g tissue (with denervation), and total naringenin was 0.26±0.04 nmol/g tissue (sham: without denervation) and 0.23±0.02 nmol/g tissue (with denervation) (Table 2). These results correlated with ones of the experiment without a blood reflux under identical experimental conditions (experiment II). These data suggested that prenylation dramatically increased naringenin accumulation in skeletal muscle. The content of the aglycone in GM was 0.35±0.09 nmol/g tissue (sham: without denervation) and 0.50±0.06 nmol/g tissue (with denervation) (Table 2). 8-PN accumulated in muscle tissue mostly as its conjugated metabolites (although its aglycone also accumulated at a low level).

### Cellular Uptake of 8-PN in Mouse C2C12 Myotubes

We calculated the logarithm of the *n*-octanol/water partition coefficient (i.e., *C*logP) of 8-PN and naringenin as an index of their hydrophobicity. We measured their uptake in mouse C2C12 myotubes to understand the reasons for the enhanced accumulation by prenylation of naringenin. Prenylation increased the hydrophobicity of naringenin because the *C*logP of 8-PN and naringenin were calculated to be 4.40 and 2.44, respectively. 8-PN was incorporated into cells and/or associated with cellular membranes at a much higher level than that observed in naringenin, and remained in cells at a constant level until 24 h of incubation (Figure 4). These results suggested that prenylation of naringenin accelerated cellular uptake and inhibited the excretion of naringenin from mouse C2C12 myotubes. The resulting higher accumulation in the cell and/or association with cell membranes could be correlated with an increase in hydrophobicity due to prenylation.

### Blood Circulation of 8-PN in Mice

We investigated the plasma concentration and pharmacokinetics of 8-PN and naringenin as an index of their bioavailability by stomach intubation of these flavonoids in mice. Plasma concentrations of total 8-PN and total naringenin were obtained by HPLC analyses with UV detection by deconjugation treatment. In contrast with muscular accumulation, the values of the maximum concentration achieved after dosing (C_max_) and area under the curve (AUC) for 8-PN were significantly lower than those for naringenin (Figure 5 and Table 3). This finding suggested that the bioavailability of 8-PN was lower than that of naringenin for a single dose. Nevertheless, the plasma concentration of 8-PN at 24 h (2.6±l.5 µM) was higher than that seen with naringenin (0.46±0.28 µM) (Table 3). Moreover, the denervation model in mice showed that 8-PN was present in plasma at 38.5±2.7 µM mostly as its conjugated metabolites after mice were continuously fed an 8-PN-containing diet for 22 days, whereas naringenin was not detected in plasma under identical conditions (Table 2). This result suggested that the plasma concentration of 8-PN was increased gradually during continuous consumption, and was different to that seen with non-prenylated naringenin.

### Preventive Effects of *H. lupulus* Powder on Disuse Muscle Atrophy

We used *H. lupulus* powder as a source of prenylated flavonoids (including 8-PN). A mixed diet of *H. lupulus* powder (5% (*w/w*) in the diet) was given to mice for 14 days before denervation. Dietary intake of *H. lupulus* powder significantly suppressed the loss in muscle weight (Figure 6).

## Discussion

Disuse muscle atrophy is an urgent public healthcare issue in aging societies because it is closely associated with the bedridden state [33]. However, medications for the relief of this condition are not available. Our previous study [1] revealed that periodic injection of the flavonol-type flavonoid quercetin into the GM was effective for prevention of the loss of muscle weight in mice that underwent tail suspension (which was used as a model of disuse muscle atrophy). Therefore, skeletal muscle is one of the target tissues of flavonoids for exerting their pharmacological functions. We first demonstrated that a prenylflavonoid prevented the loss of function of the GM in mice that underwent sciatic denervation (which is an alternative model of disuse muscle atrophy) (Figure 2). The prenylflavonoid 8-PN was administered orally to mice by mixing with the diet for 18 days before denervation. Hence, 8-PN was assumed to prevent disuse muscle atrophy after intestinal absorption and transport into the target muscle tissue. It can be deduced that prenylation increases the bioavailability of a continuously ingested flavonoid and promotes its accumulation in muscle because the plasma concentration of total 8-PN and its content in the GM were much higher than those of naringenin (Table 2). Enhancement of accumulation in muscle tissue is how flavonoids exert preventive effects upon disuse muscle atrophy. Several reports have shown that orally administered naringenin is mostly metabolized into conjugated metabolites in intestinal epithelial cells and hepatic cells before entering the blood circulation, resulting in quite low (or undetectable) levels of its aglycone in plasma [34], [35], [36]. In general, conjugation reactions are known to diminish a wide variety of the biological activities of flavonoids because conjugated metabolites are characterized as hydrophilic, stable products to be excreted into urine [37]. One study using quercetin indicated that all tissues (including muscle) contained 30–100% of the deconjugated aglycone of total flavonoids after long-term supplementation of quercetin to pigs [38] even though the aglycone could have been derived from deconjugation during the extraction procedure. In contrast, neither the GM nor plasma accumulated naringenin in the present study. Interestingly, herein we demonstrated that a considerable amount of the 8-PN aglycone was present in the plasma and GM. In particular, the content of the 8-PN aglycone was almost identical to that of total naringenin in the GM (Table 2). The ratio of the aglycone to total 8-PN in the GM was 0.15 without denervation and 0.17 with denervation, and was nearly tenfold higher than that in the plasma (0.016). Therefore, it is plausible that *in situ* deconjugation of 8-PN precedes its accumulation in the GM for it to exert preventive effects. Some authors have argued that deconjugation of dietary flavonoids must occur for these compounds to exert their functions *in vivo* [39], [40], [41]. Further studies are required to clarify the role of deconjugation in the preventive effects of prenylated flavonoids.

We selected 8-PN for evaluation of the structural significance of prenylation in dietary flavonoids for exertion of their physiological functions. Binding of the prenyl group to the molecule should accelerate intestinal absorption by passive diffusion because such binding increases lipophilicity and allows the molecule to have a higher affinity to cellular membranes [42]. High accumulation of 8-PN in muscle tissue and the appearance of preventive effects may be derived from its high bioavailability based on effective absorption in the intestine. However, the time-courses of the plasma concentrations of 8-PN and naringenin in a single dose (Figure 5) and their values of C_max_ and AUC shown in Table 3 indicated that the prenylation of flavonoids lowered their effectiveness with regard to intestinal absorption and blood circulation. Pang et al. [43] reported that a prenylated chalcone exhibits little efflux into the basolateral side when incorporated into Caco-2 cells (a cellular model of intestinal absorption). It is therefore likely that prenylation suppresses the intestinal absorption of flavonoids by interfering with efflux into the portal vein. In contrast, the plasma concentration of total 8-PN 24 h after administration was significantly higher than that seen with naringenin (Table 3), indicating that prenylation reduces the rate of elimination into urine. This may be why 8-PN was present in plasma at a considerable level after its ingestion for 22 days (Table 2). Our study on uptake using cultured mouse C2C12 myotubes (Figure 4) strongly suggested that prenylation significantly enhanced the accumulation of flavonoids within cells and/or their association to cellular membranes in mouse C2C12 myotubes because it increased passive transport into cells and interaction with cellular membranes owing to increased lipophilicity. Therefore, prenylation seems to be an effective tool to improve the physiological functions of dietary flavonoids by increasing the concentration to target tissues through a reduction in the rate of elimination from the circulation and higher uptake into cells.

Results from studies on denervated mice demonstrated that 8-PN suppressed atrogin-1 content in denervated muscle compared with control-diet mice (Figure 2c). 8-PN is known to be a powerful phytoestrogen [44], [45], [46]. Some studies suggest that estrogenic status affects recovery from disuse muscle atrophy by accelerating the Akt signaling pathway [22], [23]. That is, estrogen deficiency inhibits the reproduction of muscle, and estradiol supplementation accelerates the regeneration of skeletal muscles. Our results clearly indicated that 8-PN intake accelerated Akt phosphorylation (Figure 2c). Taken together, the mechanism for 8-PN-dependent prevention of muscle atrophy may be related to its estrogenic activity, which can promote post-natal growth and delay the degradation of skeletal muscles. Insulin-like growth factor-1 (IGF1)/phosphatidyl inositol 3-kinase (PI3K)/Akt pathway-mediated phosphorylation of FoxOs by phosphorylated Akt causes their exclusion from the nucleus, thereby preventing their transcription activity [26], [29]. It is therefore likely that 8-PN suppresses the expression of atrogin-1 by inhibiting the transcription activity of FoxOs by elevating the phosphorylated Akt level. 8-PN may also induce protein synthesis because hypertrophy in skeletal muscle was also shown to be activated by the IGF1/PI3K/Akt pathway [47]. Further study is required to clarify exact mechanism for the preventive effects of 8-PN on disused muscle atrophy.

8-PN is a demethylated metabolite of isoxanthohumol through the CYP450 reaction in the liver [14]. This prenylated flavonoid can also be produced from isoxanthohumol by the action of microflora in the digestive tract [15], [16] Isoxanthohumol is produced from xanthohumol *via* a ring-closing reaction during brewing [12], [13]. Therefore, xanthohumol and isoxanthohumol present in *H. lupulus* and beer can act as pro-8-PN. Stevens et al. showed levels of isoxanthohumol and 8-PN in beer to be 40–3,440 µg/L and 1–240 µg/L, respectively [12]. It has been shown that, in healthy post-menopausal Caucasian women, nearly 25% were moderate producers and 15% were strong producers of 8-PN based on urinary excretion through microbial conversion capacities [15]. A clinical study demonstrated that orally administered 8-PN was absorbed rapidly at a very limited level and was associated with decreased concentrations of luteinizing hormone in post-menopausal women [36]. Therefore, diets or supplements containing 8-PN (or an 8-PN precursor) could be beneficial for human health. *H. lupulus* is known to contain isoxanthohumol and 8-PN at 0.1–1.0% [12]. The results regarding intake of *H. lupulus* (Figure 6) could suggest the beneficial effects of *H. lupulus* on the prevention of disuse muscle atrophy. Bolca et al. found that 8-PN could accumulate in human breast tissue if 8-PN producers were given *H. lupulus* for 5 days [48]. Our results in mice suggest that daily consumption of 1150–1250 nmol 8-PN/kg bw/day (0.4 mg/kg bw/day) is effective for the prevention of disuse muscle atrophy. For an individual weighing 50 kg, this corresponds to daily consumption of 1 kg dry weight of *H. lupulus* and 83–20,000 L of beer. However, continuous consumption of 8-PN or its precursors in food and beverages every day may lead to a gradual increase in 8-PN content in target tissues. It is likely that daily consumption of foods or beverages containing 8-PN or its related compounds could contribute to the prevention of disuse muscle atrophy, although the study was limited to in model animal experiment using denervated mouse.

In summary, prenylation enabled naringenin to prevent disuse muscle atrophy in denervated mice. Prenylation also significantly enhanced accumulation in target tissues. These findings strongly suggest that prenylation of flavonoids induces their preventive effects by increasing their contents in target tissues. Prenylation seems to be a promising tool for developing and enhancing the pharmacological and physiological functions of dietary flavonoids.
